# Targeting the entry step of SARS-CoV-2: a promising therapeutic approach

**DOI:** 10.1038/s41392-020-0195-x

**Published:** 2020-06-17

**Authors:** Jing Li, Peng Zhan, Xinyong Liu

**Affiliations:** Department of Medicinal Chemistry, Key Laboratory of Chemical Biology, Ministry of Education, School of Pharmaceutical Sciences, Shandong University, Ji’nan, 250012 China

**Keywords:** Cell biology, Infectious diseases

**Fig. 1:**
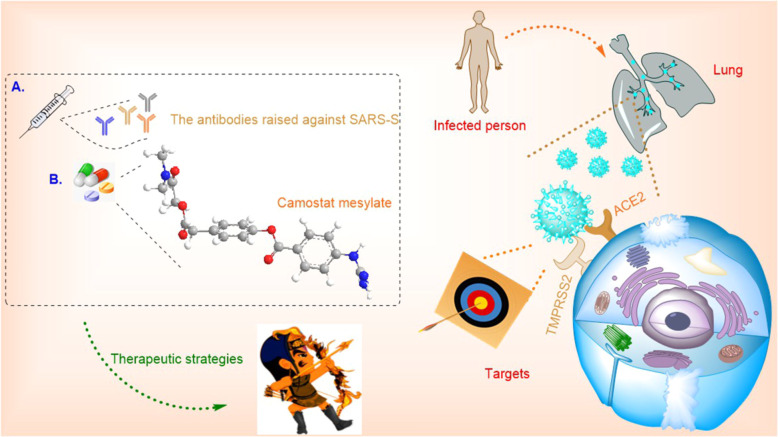
Entry of SARS-CoV-2 into host cells depends on ACE2 and TMPRSS2, and effective treatment strategies can prevent this process. For example, **a** Antibodies raised against SARS-S could cross-neutralize SARS-2-S. **b** Camostat mesylate inhibits TMPRSS2 and interferes with this process.

A very recent study published in *Cell* by Hoffmann and coworkers identifies the key proteins exploited by SARS-CoV-2 to invade cells, and finds some effective therapeutic strategies that can block cell invasion. These findings should facilitate work on novel therapeutic strategies to overcome SARS-CoV-2 infection (Fig. 1).^1^

Currently, COVID-19, which is caused by SARS-CoV-2, is rapidly spreading in humans, posing a global health emergency (https://www.who.int/); as of April 29, 2020, there were 3,018,681 confirmed cases and 207,973 deaths. Understanding the receptor recognition mechanism of the coronaviruses, which adjusts its pathogenesis, transmission rate, and host range, is the key to overcome the epidemic.^1,2^ The S protein of coronaviruses is essential for the virus to invade cells. Furthermore, entry requires cellular proteases to prime S protein; they cleave the S protein at the S1/S2 and S2′ sites, which facilitates fusion of the viral and target cell membranes that are mediated by the S2 subunit. All of us know that SARS-CoV takes angiotensin-converting enzyme 2 (ACE2) as its entry receptor and uses the cellular serine protease TMPRSS2 to prime S protein.^3,4^ The amino acid homology between SARS-2-S and SARS-S is ∼76%,^1^ but how SARS-CoV-2 entry remains to be fully explored.The amino acid homology between SARS-2-S and SARS

In order to further understand the mechanism of viral entry, Hoffmann et al. first looked for evidence of valid proteolysis of SARS-2-S. Immunoblot analysis of SARS-2-S protein expressed by 293T cells with C-terminal antigen tag exhibited a band of S2 subunit, suggesting that SARS-2-S can be effectively hydrolyzed, in accordance with the existence of several arginine residues in its S1/S2 cleavage site. Interestingly, the zoonotic potential of coronavirus is determined by the cleavage site sequence of S protein.^1^ Therefore, further studies are needed to see whether the invasion of SARS-CoV-2 on human cells also requires a multibasic cleavage site, and to characterize the cleavage site(s) in detail.

Next, the authors used VSV virus bearing SARS-2-S and SARS-S to infect a series of human and animal cell lines, and observed that they invade an identical cell pedigree. In line with this finding, most of the amino acids essential for the binding of ACE2 and SARS-S are conserved in SARS-2-S, and the directed expression of ACE2, rather than the human DPP4 or human aminopeptidase N, the entry receptor of MERS-CoV and HCoV-229E respectively, permitted SARS-CoV-2 and SARS-CoV to successfully infect insensitive BHK-21 cells. Furthermore, antiserum raised against human ACE2 could protect BHK-21 cells from the invasion of SARS-CoV-2 and SARS-CoV. Collectively, these studies strongly implicate that ACE2 is the cellular receptor of SARS-CoV-2. In addition, the three-dimensional structure of the complex of SARS-2-S with ACE2 has been parsed,^2,5^ which lays a solid foundation for the studies of vaccines, antibodies, and drugs. It is noteworthy that SARS-CoV can target ACE2 that is distributed in extrapulmonary tissues,^1^ but whether SARS-CoV-2 behaves in the same way remains to be established.

Subsequently, the authors explored the protease dependence of SARS-CoV-2 entering cells. The endosomal cysteine proteases cathepsin B and L (CatB/L) and TMPRSS2 could prime SARS-S, and among these, TMPRSS2 is indispensable.^4^ To investigate the involvement of SARS-2-S, the authors initially evaluated the roles of TMPRSS2 and CatB/L separately. Treatment with ammonium chloride, which hinders CatB/L activity by elevating endosomal pH strongly, inhibited the entry of SARS-CoV-2 and SARS-CoV into TMPRSS2^–^ 293T cells, but had lower efficiency in inhibiting the entry of the viruses into TMPRSS2^+^ Caco-2 cells. Camostat mesylate, a TMPRSS2 inhibitor that has been approved in Japan, could partially prevent the virus from entering Caco-2 cells, but had no effect on 293T cells, while E64d, the inhibitor of CatB/L, had the opposite effect. Importantly, the invasion of the virus was completely suppressed when camostat mesylate and E64d were simultaneously added, indicating that both CatB/L and TMPRSS2 are all necessary for the SARS-2-S priming. In addition, targeted expression of TMPRSS2 protected SARS-CoV-2 from inhibition by E64d, which further confirmed that TMPRSS2 can prime SARS-2-S. Further study found that lung cell infection was consistent with these findings. Intriguingly, the antibodies against SARS-S could cross-neutralize SARS-2-S, suggesting that increasing the antibody response to SARS-S in the period of infection or vaccination could alleviate SARS-CoV-2 infection.

Overall, this study reveals an essential commonality between SARS-CoV-2 and SARS-CoV infections, which may translate into similar infectivity and disease pathogenesis. Moreover, this work establishes targets for antiviral intervention, such as the host protein TMPRSS2, providing a basis for finding broad-spectrum drugs, to which resistance might not readily develop. Camostat mesylate, an inhibitor of TMPRSS2, showed some inhibitory effect on viral infectivity, and it is currently undergoing clinical trials in Denmark. This work also offers clues to other potential therapies, such as antibodies, for treating COVID-19.
